# The Fate of Pollutants in Porous Asphalt Pavements, Laboratory Experiments to Investigate Their Potential to Impact Environmental Health

**DOI:** 10.3390/ijerph14060666

**Published:** 2017-06-21

**Authors:** Susanne M. Charlesworth, Jamie Beddow, Ernest O. Nnadi

**Affiliations:** 1 Centre for Agroecology, Water and Resilience, Coventry University, Coventry CV1 5FB, UK; 2 Health and Life Sciences, Coventry University, Coventry CV1 5FB, UK; aa0412@coventry.ac.uk; 3 Hidrolef, Department of Environmental Sciences, Federal University of São Carlos, Sorocaba, Sao Paulo-SP-264, CEP 18.052-780 Brazil; ernest_nnadi@yahoo.co.uk

**Keywords:** Sustainable Drainage System (SuDS), porous paving system (PPS), porous asphalt (PA), contamination, heavy metals, hydrocarbons, rainfall simulation

## Abstract

Pervious Paving Systems (PPS) are part of a sustainable approach to drainage in which excess surface water is encouraged to infiltrate through their structure, during which potentially toxic elements, such as metals and hydrocarbons are treated by biodegradation and physical entrapment and storage. However, it is not known where in the PPS structure these contaminants accumulate, which has implications for environmental health, particularly during maintenance, as well as consequences for the recycling of material from the PPS at the end-of-life. A 1 m^3^ porous asphalt (PA) PPS test rig was monitored for 38 months after monthly additions of road sediment (RS) (367.5 g in total) and unused oil (430 mL in total), characteristic of urban loadings, were applied. Using a rainfall simulator, a typical UK rainfall rate of 15 mm/h was used to investigate its efficiency in dealing with contamination. Water quality of the effluent discharged from the rig was found to be suitable for discharge to most environments. On completion of the monitoring, a core was taken down through its surface, and samples of sediment and aggregate were taken. Analysis showed that most of the sediment remained in the surface course, with metal levels lower than the original RS, but higher than clean, unused aggregate or PA. However, even extrapolating these concentrations to 20 years’ worth of in-service use (the projected life of PPS) did not suggest their accumulation would present an environmental pollution risk when carrying out maintenance of the pavement and also indicates that the material could be recycled at end-of-life.

## 1. Introduction

It is well known that hard, impermeable surfaces lead to flooding and contamination and that Sustainable Drainage (SuDS) approaches integrated into both building and road construction reduce flood risk [1]. Porous paving systems (PPS) are an efficient attenuation mechanism for storm water flow across hard surfaces such as car parks and pedestrian areas [2]. They also have the potential for remediation of some pollution in situ and whilst much is known about the development of biofilms in association with block paver (BP) PPS and their pollution remediation role in terms of hydrocarbon biodegradation [3], there has been very little information on the removal of storm water pollutants specifically in Porous Asphalt (PA) PPS. They can also provide a reservoir for storage of water prior to infiltration or conveyance elsewhere [4].

Much of the research on PA has focused on noise and traffic spray reduction of road surface overlays [5]. Of the research into the pollution retention and infiltration properties of PPS in general (i.e., a full depth infiltrating pavement), much has concentrated on BP [6], and whilst the pollutant trapping efficiency of PPS is well known [7], interest has mainly been in the possible reduction of the infiltration capacity of the surface course due to blocking or clogging by sediment (e.g., [8]). What is not known presently is where physically in the structure the particulate associated pollutants (PAPs), remain. In fact, [2] specifically highlight the importance of this aspect of SuDs research by stating that both long- and short-term impacts of the pollutants which remain trapped in the PPS should be investigated.

Knowledge of the fate of PAPs in PA PPS has many important implications including potential impacts of released contaminants for the environment in general and possibly human health during maintenance procedures. Detailed research has been carried out on the remediation of pollutants such as metals and hydrocarbons by BP PPS by many researchers, and this has been reviewed by [2,9,10]. Geotextile is generally recommended for BP PPS and this was found by [3,6] to be the site of the development of a biofilm which both biodegraded hydrocarbons and trapped PAPs. A geotextile is generally not used in PA PPS, and thus the site of development of any biofilm, which would be related to the numbers of microorganism colonies found, is largely not known. However, biofilms tend to develop wherever there is a suitable surface in contact with water such as pipes, sediment and stones on river beds. Biofilms in this context are well-organised micro-ecosystems of bacteria, fungi, and protists living in an extracellular polymeric substance (EPS), which utilize the hydrocarbons as a source of nutrients [9]. The EPS is secreted by the biofilm, holding it together and protecting the organisms contained in it from environmental stress and xenobiotic substances such as pesticides and antimicrobials. It is a glycocalyx, containing the following anionic groups: proteins, polysaccharides, humic substances, nucleic acids, phospholipids, and other polymers [11] which are present associated with uronic acid and proteins; and the following cationic groups: carboxyl, phosphoric, amine and hydroxyl ionisable functional groups in association with amino acids [12]. The biofilm therefore carries a charge via the functional groups allowing it to sequester toxic metals, minerals, and nutrients from the surrounding liquid [13]. Studies which have investigated the metal-binding capacities of EPS have suggested that a small amount of it can bind a relatively large amount of metals [14]. The cell walls of bacteria are also capable of providing binding sites for metals and [15] found that metals such as Cd^2+^, Ni^2+^, and Zn^2+^ in wastewater were bound to microbe cell surfaces. This binding of the pollutants does not mean that they are treated; metals in particular do not biodegrade and thus these contaminants can accumulate in the PPS structure. To prevent clogging, PA PPS is usually cleaned by means of vacuuming to remove any sediment; it is possible that any associated metals may be released, particularly if conditions, such as pH or Eh, change, which can be transported to other locations, causing environmental degradation.

The aims of this study were therefore twofold: firstly, to use a laboratory-based PPS test rig with a PA surface course to investigate the ability of the PPS to treat applied pollutants (Road Sediment (RS) and unused oil). Secondly, to ascertain the fate of the applied contaminants in the test rig structure. This will enable an assessment to be made of the treatment capability of the PA PPS and also at its end-of-life, or should major refurbishment be required, knowledge of the pollutant profile would enable suitable disposal and recycling of the material removed to take place, which would add to the sustainability credentials of PA PPS.

## 2. Materials and Methods

The methodology reflects the fact that the study was undertaken in two stages: the first stage was to monitor the PPS test rig’s efficiency to treat applied pollutants at monthly intervals over a period of 38 months. The second stage involved a coring exercise to investigate where in the test rig structure any remaining pollutants were found.

### 2.1. Monthly Monitoring to Assess Pollutant Treatment

**Figure 1:**
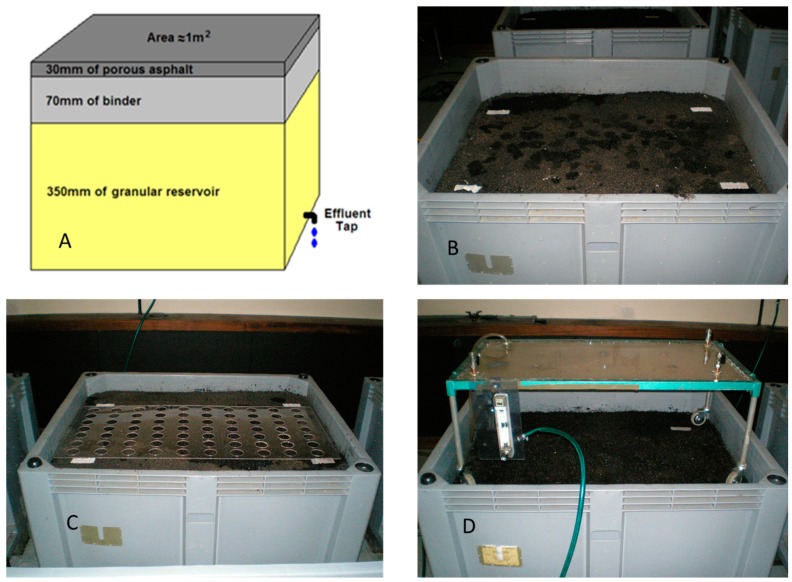
(**A**) Structure of the test rig; (**B**) surface course during monitoring; (**C**) Perspex quadrat in place; (**D**) rainfall simulator used during monitoring.

Whilst in total, 9 individual 1 m^3^ PPS test rigs were constructed for the project, including 6 with PA surface courses, 2 with porous concrete and 1 with BP, only the results of monitoring of the cored PA test rig are reported here. Figure 1A shows the structure of the PA test rig chosen to be cored, with Figure 1B showing the rig during the monitoring work. The box was made of plastic with a drainage tap in the base for collection of the effluent.

Two test pollutants were used: RS and clean/unused engine oil. Unused engine oil was chosen as its chemistry was likely to be more homogenous than used oil and hence would give comparable results throughout the monitoring study. The RS was collected in bulk from Coventry’s CV1 street cleansing depot; coarse material such as paper and stones were removed and it was dried, homogenised, and screened to <0.5 mm on return to the laboratory.

A clear Perspex quadrat was made to fit the surface of the test rig to identify where the pollutants were to be applied (Figure 1C). Seventy holes (5.0 cm in diameter) were drilled into the quadrat in a grid and each hole numbered. Tables of random numbers were used to identify where on the quadrat the pollutants were to be applied. For the first 3 months of the experiment, no pollutants were added in order to establish background concentrations in the effluents, and also to flush out any pollutants already contained in the rig. After the initial 3 months, the oil and RS were applied to the test rig surface at monthly intervals at rates of 25 mL/m (using a calibrated syringe) and 21 g/m respectively. The oil volume was well in excess of that reported by [16] as typically associated with storm water runoff (<0.1 g/L) and the RS used was double that reported by [17] of up to 12.6 g/m from a “dirty” road; this value has also been used in previous laboratory simulation experiments [18]. At the end of the monitoring period, a total of 367.5 g RS and 430 mL oil had been applied, thus representing a worst-case scenario, probably reflecting a highly contaminated industrial or heavily trafficked site. Following addition of the pollutants, the surface was artificially rained on at monthly intervals using the rainfall simulator shown in Figure 1D at a rate of 15 mm/h for 52 min (13 mm water in total) for the equivalent of 38 months’ service. Approximately 500 mL of the discharged effluent water from each rainfall event passing through the rig was collected. Between 150 and 200 mL was collected three times: the beginning of outflow (after 15–30 min), midway (after 60 min), and at the end (after 240 min). Samples were collected in clean 500 mL screw top containers and stored at −20 °C prior to analysis.

### 2.2. Coring Exercise Methodology

**Figure 2:**
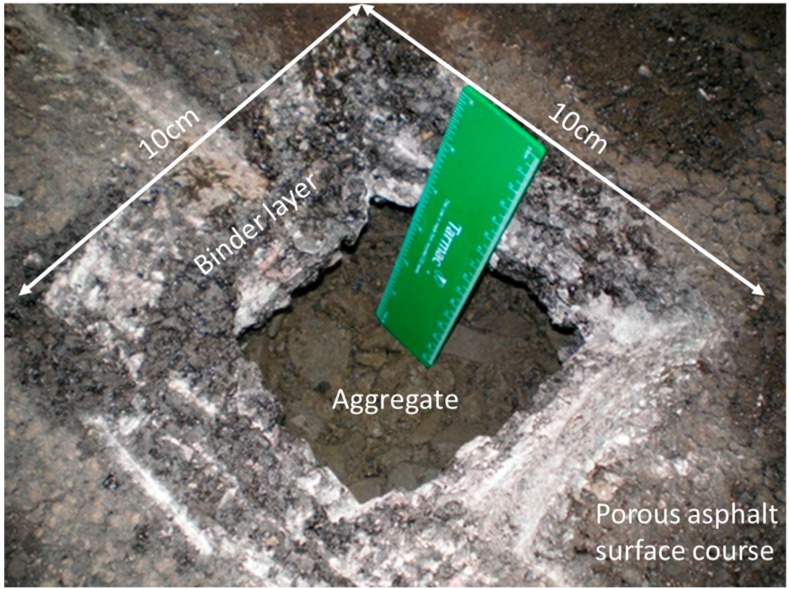
Hole excavated in the surface course of the test rig to enable access to the layers beneath.

Since drilling down through the surface of the PA test rig was thought to generate too much dust, a hole 10 cm × 10 cm was excavated in the surface and binder course by hand using a hammer and chisel as shown in Figure 2. Dust and debris from the cutting procedure was regularly vacuumed away to reduce contamination of lower levels with material from above. Individual pieces of asphalt/binder course, aggregate, and any loose sediment were then carefully removed by hand, their depth noted, and the samples stored in labelled plastic bags and refrigerated or frozen prior to further analysis. Samples of loose materials from the surfaces of the aggregate were removed using a plastic spatula and weighed. Samples were obtained according to the structure of the PPS from the surface and binder courses down through the various levels of aggregate and into the base layer at a depth of 40–45 cm.

### 2.3. Analytical Methodology

The <5 mm RS was digested using a standard wet acid technique in a microwave, followed by analysis for a selection of heavy metals using a Perkin Elmer 5300 DV ICP-OES (Perkin Elmer, Waltham, MA, USA) [19]. To ascertain the metal content of the unused oil, 1 g was mixed with 50 mL of deionised water and shaken overnight. This mixture was then filtered through a Whatman No. 1 filter paper to remove excess oil prior to ICP analysis. Total acid digestion was used to compare heavy metal concentrations in RS with published background values [20], ICRCL (International Committee on the Redevelopment of Contaminated Land) Trigger concentrations [21] and CLEA SGVs (Contaminated Land Exposure Assessment Soil Guideline Values) [22] to contextualise the results with guidelines on what would be considered contaminated.

The effluents collected from the test rigs were tested for heavy metals (Cd, Cu, Ni, Pb, and Zn), hydrocarbons (oil and grease: HC), and total suspended solids (TSS). For the metals analysis, a 100 mL aliquot of each effluent was acidified to a pH of <2 with concentrated nitric acid and metal concentrations in the resulting solution measured using ICP-OES. Total HC concentration was determined by absorbance using a Horiba OCMA 310; a 20 mL aliquot of each effluent sample was acidified with a few drops of 5 M HCl, mixed with 10 mL of S316 solvent in the analyser which then measured the concentration of dissolved HC. Total suspended solids were determined gravimetrically. Each bulk effluent sample (500 mL) was shaken well and a 250 mL subsample filtered through a 47 mm diameter GF/C filter paper which had been previously dried and weighed. The filter paper and sediment were then dried at 105 ± 5 °C for 4 h and allowed to cool in a desiccator for at least 1 h prior to re-weighing. Total suspended solids (mg/L) was calculated from the weight of the dried filter paper with and without the filtered sediment.

The leachability of the heavy metals from the material removed from the core were assessed to indicate their potential bioavailability [23] by extracting with 0.05 M EDTA at pH 7 and shaking for 1 h according to [24]. The pH was determined by suspending samples in deionised water at a water:sample ratio of 50 mL: 20 g and the pH of the supernatant liquid determined using a standard laboratory meter. In order to determine microbial presence in the test rigs, the solid material was washed with a sterile saline solution, and viable counts taken from aliquots which were serially diluted and applied to nutrient agar plates and incubated at about 20 °C for 24 to 48 h. Following incubation, the number of colonies on each of the agar plates was counted. This was a non-selective method for heterotrophs in which non-pathogenic organisms were the focus.

## 3. Results and Discussion

This section includes initial heavy metals analysis of the RS and oil, as well as results of the monitoring and coring exercise.

### 3.1. Heavy Metals in RS and Oil

Table 1A shows total mean metal concentrations found in the RS, which were compared with published background concentrations [20], CLEA SGVs [22] where available or otherwise trigger concentration [21]. Cadmium and Ni in the RS are not above background, but Cu is 10 times, Pb 5 times, and Zn 6 times their respective background values, with Cu and Zn exceeding their guidance values. These results are similar to those found in [25] where Cu and Zn were highlighted as elements of concern. Lead is of less concern now due to the introduction of unleaded petrol and a reduction in its use in paint. It appears above background due to its long residence times and hence represents historical concentrations of Pb still found in urban solid deposits [25]. Metal concentrations in the oil are also shown in Table 1A which reveals relatively high values for both Zn and Cd.

### 3.2. Long Term Monitoring of Effluent Quality

Monitoring of the test rig over 38 months indicated that very little of the applied pollutants appeared subsequently in the effluent. This suggested that both dissolved and particulate-associated pollutants as well as oil, were being effectively trapped or treated within the structure.

#### 3.2.1. Oil and Grease Determination

**Figure 3:**
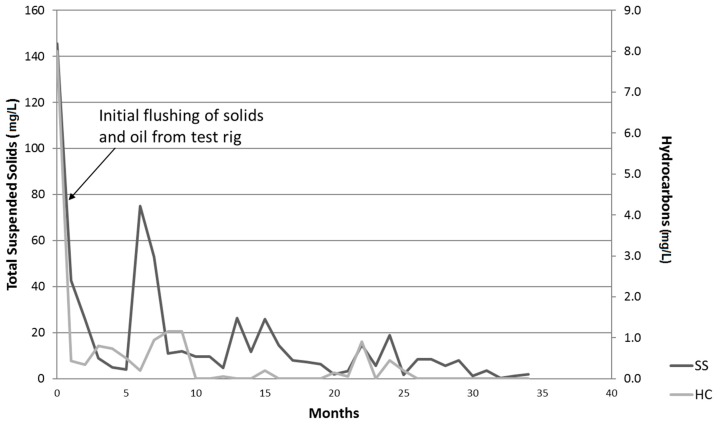
Hydrocarbons (HC) and suspended solids (SS) in the effluent (mg/L).

The results of hydrocarbon (oil and grease) determination showed that levels were all either below or very close to the analytical detection limit of 1 mg/L, with little indication of increasing concentration (Figure 3). Over the course of the monitoring programme, more than 430 mL of oil was added; in total, less than 0.5 mL of oil was detected in the effluent indicating that almost 99.9% of the added oil was retained in the PPS structure. There was no indication of any breakthrough of oil even after being subjected to such high loadings for 3 years.

#### 3.2.2. Total Suspended Solids

Other, similar studies of TSS in PPS range between 10 and 20 mg/L [27,28] with typical mean levels in effluents required for consent to discharge ranging between 30 and 100 mg/L dependent on site and industrial process. As can be seen in Figure 3, mean levels for the present study fall well within these ranges, with higher levels measured earlier in the study probably due to loose particles being washed off the rig component materials as the monitoring began.

#### 3.2.3. Metals in the Artificial Rainwater and Effluents

**Figure 4:**
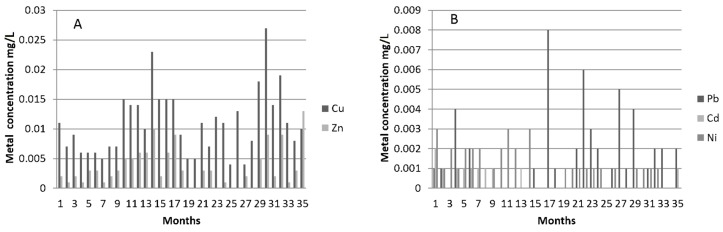
Metal effluent quality (**A**) Cu and Zn (**B**) Pb, Cd, Ni (mg/L).

There were relatively high concentrations of elements such as Cu and Zn present in the tap water used for the artificial rainfall (see Table 1). However, metal concentrations found in the effluents were consistently lower indicating the PA pavement’s ability to treat dissolved metals. There was also no indication of any deterioration in effluent quality during the later stages of the monitoring program. As can be seen in Figure 4, the metal concentration of most samples was well below the WHO drinking water guidelines shown in Table 1 [26].

Whilst it is accepted that the water discharged from PPS is unlikely to be drunk directly, comparing against [26] potable water guidelines provides a best-case scenario against which the quality of the effluent can be evaluated. The quality of water to be discharged into the environment depends on the status of the receiving waterbody, or the use to which that water is being put. Comparators are therefore site specific, for example, it may be that the water will be abstracted for drinking water or it may be discharged into salmonid rivers which have highly specific regulations regarding water quality determinands. In fact, [26] states that if national standards for water quality regulations are not stringent enough then the drinking water guidelines should be referred to, particularly in catchments used for the abstraction of drinking water. EQS Directive 2008/105/EC and the WFD 2000/60/EC also argue that catchments used for the abstraction of water should be as close to standards suitable for drinking water as possible to save on treatment costs. If the downstream environment is known, then specific comparators can be used, but for the purposes of this laboratory-based study, [26] values were used.

**Figure 5:**
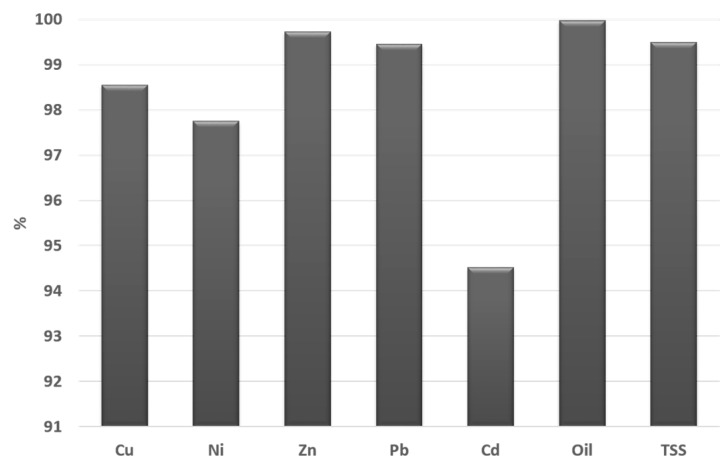
Retention of pollutants in the PA test rig as a percentage of the amount added.

Figure 5 shows the percentage of each pollutant retained; this figure is based on estimates of the total pollutant loading from measurements of the concentrations in RS, oil and the artificial rain water (see Table 1) compared with the total pollutant output in the effluents. As can also be seen from Table 1, a significant amount of Cu and Zn was applied to the models in the artificial rainwater feed and Zn is also found in high concentrations (300 mg/L) in the unused oil. In each case, more than 90% of the added load appears to have been retained within the PPS structure (Figure 5). The retention levels for Cu and Zn, the two metals added in the highest concentrations, were greater than 98%. The lowest retention was that for Cd, which even so was >94%.

The results of the monitoring study indicated, therefore, that pollutants added to the test rig surface were being effectively trapped or treated in the PPS structure. The finding that both heavy metal concentrations and suspended solids levels in the effluents from the rigs were low supports the earlier conclusion that the heavy metals in RS may be particulate-associated. After 3 years of monitoring there was no indication of any deterioration in the chemical water quality of the effluent. For the receiving environment, therefore, the PPS has a positive impact by improving water quality. However, the following sections discuss the results of the coring exercise to assess whether the contaminants remaining in the PPS may constitute a hazard to human health during maintenance and refurbishment or due to sediment entrainment in the urban environment.

### 3.3. Coring Exercise-Analysis

Based on the results of the monitoring, the second half of the project sought to determine specifically where the pollutants had become trapped, and what percentage remained.

#### 3.3.1. Oil Analysis

From a visual inspection of the samples, the only layers with any visible signs of oil contamination were the surface course and upper binder layers. No free oil films or oil odour were present in samples taken from the aggregate. The oil concentrations shown in Table 2 are measurements of the oil present in the aqueous phase of the pH extracts and do not represent the total oil loading. They simply show that there was more free oil present in the surface course than the binder layer. The samples were tested using the same methodology that was used for the effluent tests and described earlier with the exception that for metals analysis, the extracts were filtered through a Whatman No. 1 filter paper prior to measurement.

The aqueous extracts from the aggregate gave erroneously high oil readings using the IR method due to the leaching of humic compounds from the sediment which yielded a highly coloured solution. Samples from the aggregate could potentially be analysed using an alternative solvent extraction methodology to determine oil contamination. Oil results from the upper asphalt layers may also be an overestimate due to the presence of the organic binder. Whilst the results should, therefore, be treated with caution, nonetheless, they provide an indication of where in the test rig relatively higher concentrations were found.

#### 3.3.2. Microbiology

The results of total viable counts per gram of rig material are presented in Table 2. These values, expressed as colony forming units per gram, give an indication of the microbial growth throughout the depth profile of the PPS. The microorganisms present are likely to play an important and beneficial role in improving effluent quality through both biodegradation of oil and organic materials and adsorption of heavy metals [4,19]. The highest numbers of organisms were found in the lower parts of the model, in the layers of aggregate which were the wettest parts of the structure. In comparison, the much drier binder and upper aggregate layers seemed be supporting much less microbial growth. Therefore, the availability of water may well be an important factor influencing their distribution. A more comprehensive study would be required to determine any influence associated with differences in nutrient availability throughout the structures (N, P, oil). Relatively high organism numbers were also found in the surface course samples. This could be an indication of their utilisation of organic matter sourced from the trapped RS and oil. The effect of a drier environment may be counteracted by the higher levels of nutrients available.

#### 3.3.3. Heavy Metals

**Figure 6:**
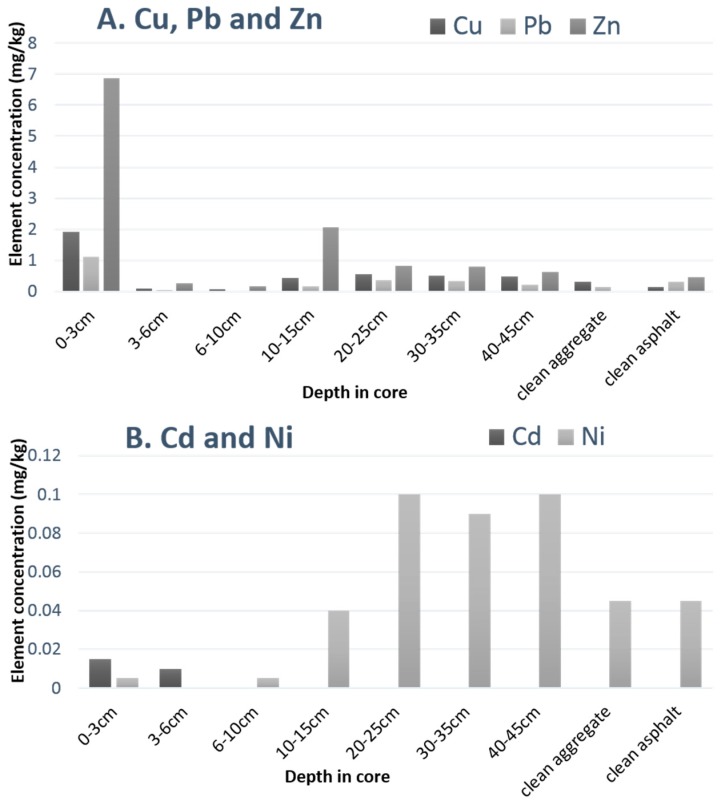
EDTA extractable heavy metals in samples of material taken from various depths within the test rig in comparison with RS, clean aggregate and clean porous asphalt. (**A**) Cu, Pb and Zn; (**B**) Cd and Ni.

Figure 6 shows that the greatest levels of the EDTA extractable metals were present in samples of material taken from the surface course. Visual inspection supported the fact that the surface course was also the greatest sink for the oil. It is likely that some of the added street dust was trapped at the surface, possibly in association with the oil, however attempts to recover the sediment were limited by its adherence to the oil.

As shown in Figure 6, the sediment that was recovered from the surface course contained higher levels of heavy metals, in particular Zn, Cu, Cd, and Pb. However, all these metals were also present, although not in such high concentrations, in the aggregates below 10 cm. The highest concentrations of Ni, however were found below 10 cm in the aggregate layers. Cadmium was under the limits of detection below 6 cm in the core. Figure 6 also shows the low levels of all metals in clean aggregate and PA contextualising their accumulation particularly in the surface and binder courses. Nickel was present in comparatively high concentrations in both the clean aggregate and asphalt. In comparison with EDTA-extracted metals in fresh RS (Table 1B), the highest concentrations from the core (Figure 6) were far less, the ratio of Cd in the core compared to fresh RS was 0.044, Pb 0.015, Zn 0.055, Cd 0.054, and Ni 0.15. This may be due to the migration of some of the heavy metals (soluble or particle-bound) throughout the test rig, of which the core samples are a small amount of the total volume. However, it does reflect % “retention” as illustrated in Figure 5, which should perhaps be renamed “% lost”. In terms of an estimate of whether these concentrations constitute a hazard, however, there is no evidence of this after 3 years’ worth of pollutant addition, bearing in mind that what was added represented very much a worst case scenario. The in-service life of PA pavements should be approximately 20 years [29], possibly longer and future work should be to examine PAP accumulation when such a pavement is refurbished, removed, or replaced. However, unlike the repair of impermeable asphalt surfaces, opportunities to examine PA refurbishment do not arise often.

The highest sediment levels were found throughout the aggregate layer. The sediment found here does not appear to be RS that has migrated through the upper asphalt layers as it was a different colour and contained different heavy metal levels in comparison with RS. It is more likely therefore, to be the original sand and mud associated with the materials used in constructing the models.

Heavy metals were found distributed throughout the full depth of the aggregate layer. The higher levels of Cu and Zn observed fits with the input of these metals into the models via the rainwater feed, and may have been bound to the sediment found in association with the lower aggregate layer. However, these metal concentrations are all well below the original RS levels as shown in Table 3, so it is unlikely that the RS migrated down through the structure, but, as is discussed above, it is more likely that they are bound to loose material used to construct the rigs at the start of the programme. The pH was neutral to alkaline (Table 2) with values above 9 measured in the middle and lower aggregate layers reflecting the fact that it was limestone. It is well known that metals become soluble under acidic conditions, and remain bound to the sediment at neutral and alkaline pHs, thus, unless environmental conditions change, it would be unlikely that PAPs would be released.

## 4. Potential for Impacts on Environmental Health

### 4.1. Potential Environmental Impacts

The results of the monitoring show that the PA PPS was able to treat the applied pollutants such that the effluent was of WHO drinking water quality. This would therefore represent little or no hazard to the environment should the PPS drain into surrounding soils or to receiving waterbodies such as groundwater in spite of studies such as [30] expressing concern. However, the focus of this study was to determine the eventual fate of the applied contaminants—this was achieved through the coring exercise. At a PPS end-of-life after about 20 years [29], perhaps due to wear of the surface course, clogging, or subsidence, it may have to either be repaired or even completely replaced. It has been established through this study that the surface course is the repository of Cu, Pb, and Zn, with Ni being found further down into the aggregate layer. However, the highest concentrations that were found were far less than those of the original RS as shown in Table 3. These levels represent the equivalent of 38 months of monitoring a worst case scenario in terms of the amount of applied pollutants. Even extrapolating this to 20 years assuming a constant rate of accumulation and acknowledging that metals do not degrade would not increase the concentrations to a problematic degree.

### 4.2. Maintenance, Refurbishment, Disposal and Particulate Resuspension

One of the main barriers to the use of PPS is the perception that they clog readily [30], maintenance is therefore important to prevent this. However, it involves operatives street-sweeping [31], vacuum-suctioning [32], or high pressure-washing [33] the surface. These processes may have potential environmental consequences should PAPs be entrained into the air or local watercourses where they can be transported to another milieu. Having been treated by the PA rig, even in the surface course, there would appear to be little concern from accumulated metals. Whilst it is well known that the movements of vehicles can resuspend particulates from the road surface [34,35], their relatively low concentrations would appear to suggest that again, this would not appear to be of great concern to the environment from re-entrained road dust particulates.

However, results showed that there were microorganisms, possibly present as an active biofilm, in the PA surface course, and these may well be removed during maintenance procedures. Following cleaning, therefore, there may be a time when treatment in the surface layer is sub-optimal due to the removal of the biofilm. The microorganisms are found simply in the surrounding air, previous studies inoculating the PPS with mixtures of microbes [3] found no advantage, in fact, experiments by [19] whereby the biofilm was removed using a herbicide, found that it recovered fairly rapidly. Numbers of colony forming microbes were found to be higher in the aggregate layer of the PA PPS, so it is likely that for the short time whilst the surface biofilm recovers, infiltrating pollutants could be dealt with deeper in the PPS structure, which could provide the focus for future investigations.

In the UK, in terms of disposal or recycling at end-of-life for the PA PPS, Waste Acceptance Criteria (WAC) established by the Landfill (England and Wales) Regulations 2002, set leaching Limit values for disposal of material through the Landfill Directive [36]. The WAC value [37] determines the disposal route, whether inert and non-hazardous, or consigned to a hazardous landfill site, or the possibility of reuse. Whilst specific WAC leaching was not carried out on the samples from the core, the EDTA extractions do indicate metal concentrations to be low; further testing may be useful before disposal at end-of-life, but it is possible that the aggregates could be recycled and reused rather than having to be disposed of, thus reducing the amount of virgin aggregate required upon replacement of the PPS and also space used for landfill.

## 5. Conclusions

After 3 years monitoring of the laboratory based PPS models of a worst case scenario, there was no indication that the pollutant retention capacity has been exceeded with >90% of the applied contaminants retained. The results show that the pollutants added to the test rig surface were being effectively trapped and treated within the PPS structures. This led to the concentrations of the heavy metals in the effluents remaining below WHO potable guideline levels and typically close to or below the analytical limits of detection. The finding that both heavy metal concentrations and suspended solids levels in the effluents were low agreed with the earlier conclusion that most of the heavy metals in the RS may be particulate-associated. The levels of HC in the effluents were close to or below the limits of detection, and indicated that more than 99% of the oil added to the test models was trapped and potentially degraded by the microorganisms present in the PPS models as shown in the coring exercise.

From the coring exercise, it appears that most of the polluted sediment and oil is trapped in the upper surface course layer. This may be advantageous in that this zone can be cleaned using high pressure jetting and the polluted sediment can be simultaneously removed. However, there is potential for any biofilm which has developed in the surface course to be removed during maintenance, reducing treatment opportunities. This scenario is likely to be short term, as biofilm development is fairly rapid. From the suite of pollutants monitored, there would appear to be little concern for environmental health when carrying out these procedures as EDTA extraction concentrations, reflecting potential bioavailability, were low. It was also found that the loose material associated with attrition of the aggregate layers may provide a secondary site for the binding of any dissolved metals percolating down through the structure, particularly Ni. This may have impacts when considering disposal or recycling of the aggregate after refurbishment which may require additional testing in order to ascertain final destination of the material. Microbial analysis suggests that the aggregate is also the site where high numbers of bacteria are found and which may play an important and beneficial role in the breakdown of hydrocarbons and further trapping of PAPs.

## Figures and Tables

**Table 1:** (**A**) Total concentrations of heavy metals in RS compared with published background and guideline values of heavy metals in unused oil (mg/L); (**B**) Average concentrations of heavy metals in test pollutants materials and artificial rainfall (tap water feed).

	Cd	Cu	Ni	Pb	Zn
**A**					
RS (*n* = 24) (mg/kg)	1.1	223	24.9	152	366
Soil background (mg/kg) [20]	0.62	25.8	33.7	29.2	59.8
CLEA SGVs (mg/kg) [22]	30	Nd	75	450	Nd
Trigger (mg/kg) [21]	3	130	70	500	300
Unused oil (*n* = 2) (mg/L)	1.7	2.0	0.46	0.43	300
**B**					
RS Soluble (mg/kg)	0.06	1.97	0.16	0.19	1.69
EDTA extractable RS (mg/kg)	0.34	45	0.67	66.5	128
Rain (tap feed) (mg/L)	0.001	0.277	0.002	0.01	0.105
Mean rig effluent (mg/L)	0.0003	0.011	0.001	0.0014	0.0035
WHO (mg/L) [26]	0.003	2.0	0.070	0.010	3.0 *

Nd = no data; * no health-based guidelines for Zn, the value given is based on its impact on taste.

**Table 2:** Summary of the results from tests from coring exercise.

Rig Samples	Depth (cm)	pH	Oil (mg/L)	Microbiology (CFU/g)	Sediment (g/kg)
Surface Course	0–3	7.1	13	1.6 × 10^5^	2.0 *
Upper Binder	3–6	7.6	2.6	1.0 × 10^3^	0.8
Lower Binder	6–10	7.5	1.5	1.6 × 10^3^	1.3
Upper Aggregate	10–15	8.9	ns	7.2 × 10^3^	38
Middle Aggregate	20–25	9.1	ns	2.0 × 10^5^	52
Lower Aggregate	30–35	9.1	ns	7.0 × 10^5^	48
Base	40–45	8.9	ns	2.1 × 10^7^	32

Notes: ns—not suitable for analysis; CFU: colony forming units; * underestimate of sediment in surface course (bound via oil to asphalt); “sediment” reported in g sediment per kg matrix material.

**Table 3:** Comparison of the maximum concentration of EDTA extracted metals (mg/kg) from the original RS and samples from the core.

Metal	RS	Core
Cu	45	2
Pb	66.5	1
Zn	128	7
Cd	0.34	0.015
Ni	0.67	0.1
